# Unpacking Summary Measures of Ethnic Residential Segregation Using an Age Group and Age Cohort Perspective

**DOI:** 10.1007/s10680-018-9475-3

**Published:** 2018-03-06

**Authors:** Albert Sabater, Gemma Catney

**Affiliations:** 1 School of Geography and Sustainable Development & ESRC Centre for Population Change, University of St Andrews, St Andrews, UK; 2 School of Environmental Sciences, University of Liverpool, Liverpool, UK

**Keywords:** Age group, Age cohort, Ethnicity, Residential segregation, Life course, England Wales

## Abstract

The residential segregation literature has underplayed the significance of age in shaping the ethnic compositions of neighbourhoods. This paper develops an age group and age cohort perspective as a way to unpack summary measures of residential segregation. Harmonised small area data for England and Wales (2001–2011) are used as a case study to explore the potential of this methodology for understanding better the role of age in the evolution of ethnic residential geographies. Our findings demonstrate the age-specificity of residential segregation, for both cross-sectional patterns and change over time. Levels of segregation vary among age groups and age cohorts and between ethnic groups, with a changing pattern of segregation as people age. Exploring change over a 10-year period, we observe that residential segregation decreases during young adulthood for all age cohorts, then increases during the late 20s and early 30s, and continues to increase until retirement. These trends are, for the most, consistent between ethnic groups. Our findings emphasise how residential segregation is a dynamic process with a significant life cycle component, with commonalities in residential decision-making between ethnic groups through the life course.

## Introduction

Age is arguably the most influential demographic characteristic on the evolution of ethnic residential patterns, and yet it is largely over-looked in most studies of residential segregation. While most individual-level studies of segregation control for age when comparing ethnic groups, little is known about the age group and age cohort variation in aggregate-level ethnic residential segregation. This is a surprising omission given that ethnic minorities’ residential geographies are distinct by age. It is also significant given the ageing of populations of many societies which are becoming increasingly ethnically and culturally diverse. In this sense, almost all contemporary studies of ethnic residential segregation suffer from a major limitation: they are entirely focused on the spatial patterns of minority groups *as a whole*, missing the variation in segregation levels which are likely by age groups and age cohorts.

The residential segregation literature is extensive and under continual advancement, including recent developments in methodological approaches (Wong 2016), conceptual frameworks (Crowder and Krysan 2016), and a growing empirical evidence base (Lloyd et al. 2014a; Maloutas and Fujita 2012; Massey 2016) for both pan-ethnic and detailed groups (Iceland et al. 2014). However, we still have great difficulty when evaluating and explaining the dynamics of residential segregation (Charles 2003; Lichter 2013). One key problem is that changes over time reflect the sum of changes in the life course of individuals, each of whom is at a different life stage. As time passes, ethnic groups grow older, and new cohorts become part of the population—a result of immigration and/or births. Since the composition of ethnic group populations change because of these factors, comparisons of *summary* or *global* measures (terms we use interchangeably throughout the paper) of residential segregation between two points in time include individuals in different stages of residential change.

There is some documentation of the significance of age in the segregation literature of the 1960s and 1970s, including how the life cycle events of marriage and childbearing spurred residential relocation from Black to White neighbourhoods (Edwards 1972; Taeuber and Taeuber 1965). Yet contemporary studies of residential segregation rarely take into consideration the implications of an obvious point: minority integration was theorised as a sequence of ‘invasion and succession’ in which the gradual out-movement of ethno-racial groups from settlement areas depends on their ages and stages in the life course (Rossi 1955). This age dimension has only been considered in depth in a small number of recent studies of residential segregation of ethnic minorities in Britain in the 1990s (Sabater 2010; Sabater and Finney 2014), and of ethno-racial groups in the USA in the 1990s (Marcum and Brown 2009) and in the 2000s (Winkler and Johnson 2016). An important finding from this research is that the movement of younger people is a key factor driving desegregation over time—and that this is the case across *all* ethnic groups. While this suggests that age is an important dimension of change in ethnic residential segregation, further research is needed to establish whether residential segregation at different times and contexts varies by age groups (i.e. between people who were born at different periods) and age cohorts (i.e. between people who were born in the same period), and whether there are similarities and specificities between ethnic groups.

The purpose of this paper is thus to analyse changes in the level of ethnic residential segregation using an age group and age cohort perspective as a way to unpack summary or global measures of residential segregation. In doing so, we exploit the dual significance of age in demography (age group and age cohort) for application to the study of ethnic residential segregation. We use ethnic group data for England and Wales for 2001 and 2011 as a case study for this work. As such, the results provide new insights into changes in residential segregation using the largest minority ethnic groups in the UK—Indian, Pakistani, Bangladeshi, Black Caribbean, Black African, Chinese, and Other White. More broadly, the paper considers if, while global segregation measures provide useful as summary measures for each ethnic group, they miss important and distinctive age group and age cohort trends; this has relevance to other contexts beyond the UK, for example, the rest of Europe and the USA.

The paper addresses three specific questions:

1. Are there distinctive patterns of segregation for young people compared to those in older ages?
2. Do ethnic groups show similar patterns of change in geographical spread and concentration: over time and for each age group and age cohort?
3. Do younger age cohorts of all ethnic groups represent a cohort/generational change in terms of spatial mixing?

Since our study is based in England and Wales, in the following section, we focus primarily on UK-based research to illustrate the significance of the age dimension; however, many of the more general observations between ethnic groups are also found elsewhere. We follow this with a discussion of the data requirements and methodological issues that need to be addressed in examining ethnic residential segregation with an age group and age cohort perspective. We then present our results and discuss the main findings and conclusions.

## Segregation and the Need for an Age Dimension

### Segregation in the UK Context

Over the past fifteen years, successive UK governments have gradually shifted from a multicultural policy to a community cohesion agenda (favouring a shared culture, identity and belonging) that aims to tackle the ‘self-segregation’ of ethnic groups (Cantle 2001). These debates reduced in dominance in the mid-2000s, but have once again become prominent in UK policy. Most notably, the Conservative Government undertook a major review of opportunity and integration (Casey 2016), which included substantial elements exploring the residential segregation of ethnic groups. The following year, the All Party Parliamentary Group on Social Integration published an investigation into the ‘social’ integration of immigrants (APPG Social Integration 2017).

Yet academic studies have consistently demonstrated that minority ethnic segregation has *decreased*, and that this has been a dominant trend since ethnicity was first captured in the 1991 England and Wales Census and detailed studies of geographical patterns were made possible (Catney 2015, 2016a, 2017a, b; Johnston et al. 2013, 2015, 2016a, b; Harris 2014; Simpson 2007). This reduction in residential segregation is in spite of the unfavourable economic position of many minority ethnic groups relative to the White British majority (Jivraj and Simpson 2015), and persistent discrimination towards ethnic minorities, for example in the workplace (Ashe and Nazroo 2016). In fact, although residential integration may not have occurred as quickly or simplistically as early theories of ‘invasion and succession’ suggested, decreasing minority residential segregation has been a dominant trend in the UK, as observed across censuses—between 1991 and 2001 (Sabater 2010; Simpson 2007) and between 2001 and 2011 (Catney 2015, 2016a; Simpson 2012). Almost all recent segregation studies have challenged assertions of ‘self-segregation’ using aggregate data at the small area level via indices of segregation (Catney 2016a), local and spatial measures (Catney 2017a, b) segregation typologies (Johnston et al. 2013, 2015, 2016a, b), or by exploring changes in ethnic composition between neighbouring areas (Harris 2014). One common conclusion from these studies is the notable reductions in ethnic residential segregation across the decade, including at the small area level.

Such studies, however, invariably measure ethnic residential segregation ‘as a whole’, and take no account of the fact that these observations may mask important variations by age groups and age cohorts within ethnic groups. Several individual-level studies in the UK demonstrate the significance of this gap in the literature. For instance, in a comprehensive analysis of the processes driving changes in spatial segregation using the Scottish Longitudinal Study, Bailey (2012) indicates the dominance of ageing and cohort replacement. Similarly, in a study of ethnic diversity, segregation and the social cohesion of neighbourhoods in London using Public Attitude Survey data from the Metropolitan Police Service, Sturgis et al. (2014) highlight that ethnic segregation within neighbourhoods is highly contingent upon the age cohort to which an individual belongs. This suggests that more nuanced analyses of ethnic residential segregation, which take account of demographic variation, are needed. In turn, this also raises important issues for policy.

### The Age Dimension of Residential Segregation

While it is implicit—and occasionally explicitly stated (Sabater 2010)—that global residential segregation levels are higher as a result of the young age composition of immigrants (Simpson 2007), the age dimension is rarely accounted for in explaining variation in spatial segregation. Aggregate-level studies cannot separate out the individual factors that contribute to residential segregation, however ‘there are reasons to theorise that both the ethnic mix of the neighbourhood of residence and patterns of migration in relation to ethnic mix vary for people of different ages, and through the course of an individual’s life’ (Sabater and Finney 2014: 271). If so, why is the age dimension missing in most studies of residential segregation?

One may argue that this is because in the broader urban geography literature, which has long explained change in neighbourhoods through processes of ‘invasion and succession’ (Johnston et al. 2014), selective migration of young adults is often taken to be the dominant process in residential segregation (Bailey 2012), thus leading to an age-homogeneous perspective which implicitly focuses on segregation among young adults only. Another, equally important, element is a common policy approach which sees an unwavering line of progression from immigration to residential integration (Vaughan and Arbaci 2011), underplaying the role of natural change in the growth of ethnic diversity (Finney and Simpson 2009; Simpson and Jivraj 2015). This growth of relatively young but ageing minority ethnic populations is relevant to understanding the local dynamics of Britain’s ethnic geography; age structures of recent immigrants are combined with those of second- and third-generation minority groups, and this juxtaposition of cohorts explains the maintenance or increase in residential segregation in some neighbourhoods (Catney 2016a).

**Fig. 1:**
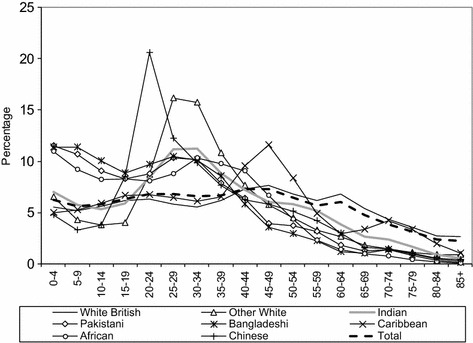
Ethnic group age structures in England and Wales 2011. *Sources*: Own elaboration using data from 2011 Census, Table KS201EW (Crown Copyright)

Since ethnic groups differ in terms of their immigration history, age structures differ between ethnic groups. The impacts of fertility and mortality rates are also different between ethnic groups, although the short- and medium-term development of minority populations in the UK is largely determined by the numerical balance between its younger and older adults (Simpson 2009). Figure 1 clearly shows that minority ethnic populations have youthful age structures compared to a mature (and ageing) White British population. While around a quarter (23.9%) of the White British population are aged between 20 and 44, more than a third (39.9%) of the non-White British ethnic groups falls into this age category. This reflects a decline in natural growth (i.e. more deaths than births) and emigration by the White British group, as well as immigration and the positive natural momentum of minority ethnic groups with relatively young age structures (Simpson and Jivraj 2015), a situation which is clearly captured by the growing populations of most minority groups (with the exception of White Irish and Caribbean) since 2001 (see Table 1). Indeed, there are also significant differences between minority ethnic groups’ age structures and growth rates, mostly due to the diversity of contemporary and historic immigration trends, including inflows of international students to UK universities (e.g. Chinese), labour migration to Britain over the past decade (e.g. the Other White ethnic group, especially Eastern Europeans via A8 Accession migration), and minority ethnic populations who immigrated during the post-war period and that are more mature demographically (e.g. Black Caribbean).

**Table 1:** 2011 England and Wales population by ethnic groups, and percentage point change from 2001. *Sources*: Own elaboration using data from 2011 Census, Table KS201EW (Crown Copyright), and complete population estimates based on the 2001 Census (Crown Copyright)

	2001		2011		Percentage point change from 2001
	Population	Percentage of total population	Population	Percentage of total population	
White British	45,721,238	87.32	45,134,686	80.49	− 6.83
White Irish	646,618	1.23	531,087	0.95	− 0.29
White Gypsy/Irish traveller	n/a	n/a	57,680	0.10	n/a
Other White	1,379,499	2.63	2,485,942	4.43	1.80
Mixed White-Caribbean	240,438	0.46	426,715	0.76	0.30
Mixed White-African	80,705	0.15	165,974	0.30	0.14
Mixed White-Asian	192,229	0.37	341,727	0.61	0.24
Other mixed	158,582	0.30	289,984	0.52	0.21
Indian	1,053,302	2.01	1,412,958	2.52	0.51
Pakistani	727,726	1.39	1,124,511	2.01	0.62
Bangladeshi	286,693	0.55	447,201	0.80	0.25
Chinese	233,346	0.45	393,141	0.70	0.26
Other Asian	247,157	0.47	835,720	1.49	1.02
African	494,668	0.94	989,628	1.76	0.82
Caribbean	572,212	1.09	594,825	1.06	− 0.03
Other Black	98,068	0.19	280,437	0.50	0.31
Arab	n/a	n/a	230,600	0.41	n/a
Other	227,497	0.43	333,096	0.59	0.16
Total	52,359,979	100.00	56,075,912	100.00	

The relatively young age structures of minority populations in the UK have two sequential impacts on ethnic residential segregation. First, natural growth (the excess of births over deaths) constitutes a major factor in expanding existing co-ethnic clusters. Second, this in situ growth is linked to housing pressure and migration out of cities, particularly from urban centres. Adjustments in housing size and tenure are a function of social and residential mobility related to employment and the family-building stages of the life course: the suburbanisation of older adults and families, in particular by those in more professional occupational classes, is well-evidenced (e.g. Catney and Simpson 2010). This movement away from ethnic ‘settlement’ areas is common to all ethnic groups (Finney and Simpson 2009; Simpson et al. 2008; Stillwell 2010) and helps explain the widespread increase in ethnic diversity and ethnic mixing across neighbourhoods (Catney 2016b). As with elsewhere, the likelihood of moving for all ethnic groups in Britain is age-dependent and peaks for the 20–24 age group (Finney 2011; Finney and Catney 2012). However, it is worth noting that for both the majority and minority groups aged under 19 or over 30, the direction of movement differs from that for young adults in their 20s. There is evidence that while families and older adults move to predominantly White British areas, young adults (aged 20–29) move to more diverse areas, driving ethnic mixing (Sabater and Finney 2014). Hence, by focusing on *subgroups* of the population, particularly age groups (Finney 2011), we gain more insight into the possible processes by which differences in ethnic residential segregation are (re)produced.

The age profile of the typical migration schedule (Rogers and Castro 1981) thus offers a potential framework to unpack summary measures of residential segregation. Because mobility is high in young adulthood and events associated with migration (ranging from education/employment to partnership/parenthood transitions) occur most frequently in this period of life, we can expect young adults to be exposed to a greater ethnic mix in their residential environments than established families and older adults (Sabater and Finney 2014). Lloyd et al. (2014b: 7) highlight the importance of this distinction in mixing across the life course for young adults’ experiences: ‘because, as they age, these young adults may have experienced more ethnic diversity than older members of their respective groups’. The example of young adults reinforces the problems of confusing the temporary—and temporal—nature of most minority settlements with permanent and enforced concentration (Peach 2005).

**Fig. 2:**
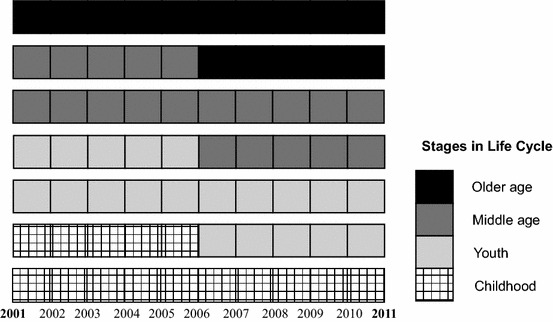
Intersection of biographic and time within the life course framework. *Source*: Own elaboration

Furthermore, this also means that global measures of residential segregation combine very different experiences of spatial integration, depending on the life stage of individuals *regardless* of ethnicity—such as childhood, youth, middle age and older age—each with its own social and residential characteristics, through which individuals pass over the course of their lives. While places with a history of settlement have consistently proved to be attractive to future immigrants (Peach 1996), neighbourhoods are unlikely to have a consistent, single demographic profile because of the complex overlapping of life course transitions over time in any place. Thus, a one-size-fits-all approach to residential segregation is problematic because it largely ignores the intersection of biographic (life course) time and historic (real) time within the life course framework. As depicted in Fig. 2, residential segregation in any neighbourhood is likely a function of a four-way intersection of key stages in the life cycle, at different points in time: childhood, youth, middle age and older age. Although most individuals (and families) do not conform to any single, uniform life cycle, the life course framework is useful in illuminating ethnic differences and similarities in patterns of residential segregation at key stages during which individuals are, for instance, leaving the parental home for work or to higher education, and from here to graduate employment, forming new families of their own, or ageing in place.

As shown in Fig. 2, another important aspect of the life course framework is that behind the intersection of biographies there is also a time or context in which individuals at different stages in the life cycle live their lives. Because time is a crucial element in the life course approach (Elder and Giele 2009), a strategy that assesses the residential experiences of recent age cohorts compared to earlier age cohorts (as opposed to age groups alone) is also important in order to evaluate whether and how ethnic residential trajectories of recent age cohorts differ from those of past age cohorts. Since changes over time in ethnic residential segregation may be the result of cohort/generational changes, it is possible that the most recent age cohorts may be following a different residential path than their predecessors. This would accord with the spatial assimilation model of socio-economic and residential integration (Johnston et al. 2007). Over time, as immigrants and, especially, their children improve their socio-economic circumstances—and as more are born in the UK (as opposed to immigration from elsewhere)—minority ethnic residential locations may diversify to less urban (suburban and rural) neighbourhoods (Catney 2016b). While spatial assimilation does not necessarily reduce segregation from Whites (as in the USA, for example: Wright et al. 2005), in the UK context the smaller predominance of the White British group in both original and new areas of immigrant settlement means that greater ethnic mixing is a likely outcome (Catney 2016a). Nonetheless, we still have limited knowledge about typical residential segregation trajectories by age cohorts of ethnic groups with varying segregation levels.

## Data and Method

Two data sources are used in this paper: Census-based Population Estimates (2001 by ethnic group and age) and Census data (2011 by ethnic group and age).^1^ The former are complete mid-2001 population estimates for sub-national areas in England and Wales including a full allowance for estimated non-response and harmonised population data for small area geographies (see Sabater and Simpson 2009), thus providing estimates for all Output Areas (175,434 units) in England and Wales by single year of age and all major ethnic groups in 2001.^2^ These are then used as building blocks for aggregation to comparable groups, ages and larger areas from the 2011 Census.^3^

There is considerable debate about what spatial unit constitutes a neighbourhood and, indeed, the importance of scale for the measurement of residential segregation, although there is no one correct scale at which to measure it (Jones et al. 2015; Lloyd et al. 2014c). The neighbourhood analysis in this paper is based on 2011 Census merged wards (hereafter wards). Using the 2011 wards can be seen as a compromise between having sufficiently large areas for small yet sufficient populations of minority ethnic groups, and avoiding areas which are so large that they miss the finer-grained spatial variability in ethnic residential patterns suggested in other work (Johnston et al. 2015; Catney 2016b). Although other studies of residential segregation use smaller, more homogeneous areas with consistent population sizes (e.g. Output Areas), these are not suitable for analyses with an ethnic group age breakdown given the significant number of areas with small numbers of ethnic groups. In addition, wards can be fully compared between 2001 and 2011 and given their electoral role, they have a functional meaning and are socially relevant; they have what Kearns and Parkinson (2001) refer to as the ‘home area’ and ‘locality’ layers of neighbourhood. In order to make the census ward boundaries consistent over time, the conversion from 2001 to 2011 Census wards has used the lowest geographical source unit possible (Output Areas) so that spatial differences in age-sex-ethnic group are respected when translating the two sets of boundaries.^4^ There are a total of 8546 wards in England and Wales, with a mean population size of 6600.

To assess the spatial situation of ethnic minorities, special attention is given to the eight largest and most stable categories from 2001 to 2011: White British, Other White, Indian, Pakistani, Bangladeshi, Black Caribbean, Black African and Chinese. Although 96% of the 2001 population that was enumerated again in 2011 reported the same or equivalent ethnic group category, there is considerable heterogeneity between the ethnic groups captured by the census (Simpson et al. 2016). The White British category showed the highest stability (with 98.8% of its 2001 population reporting again in 2011 the equivalent category), while all Asian categories (Indian, Pakistani, Bangladeshi and Chinese) were less stable (88–97%) than the White British category, but more stable than other minority categories. As such, comparisons over time may be made with the aforementioned eight categories because of their relatively high stability (Simpson et al. 2016). The use of these ethnic group categories can be viewed as a useful way to capture individual and group characteristics, predominantly in terms of (im)migration history or race.

We apply two measures of segregation: the dissimilarity index (ID) and the isolation index (P*). Although a suite of indices have been used to capture various dimensions of residential segregation (Massey and Denton 1988), we rely on the most two most commonly employed to allow straightforward comparisons of global, age group and age cohort segregation both nationally and internationally. These measures reveal the level and change over time with respect to two dimensions of spatial variation: *evenness* and *exposure*.

Residential evenness is measured with ID, which indicates how evenly people of different ethnic groups are distributed across areal units nationally or within a metropolitan area. ID is conceived here to measure an unequal geographical spread of one group relative to the rest of the population,^5^ which represents a variant of the ID also known as the segregation index (Simpson 2004). A factor of one-half is used in the computation of the index so that the index values always lie between 0 (no segregation or geographical spread through areas in the same way as the rest of the population) and 1 (complete separation). The index can be expressed as a percentage, with index values between 0 and 100. This value can be interpreted as the percentage of the ethnic group of interest that would have to move neighbourhood in order to have a distribution of the same evenness as the rest of the population. Thus, the higher the index value, the greater the unevenness. One formula for the dissimilarity index is:

1 $${\text{ID }} = 0.5 \, \times \sum\limits_{{_{i} }} {\left| {\frac{{N_{{{\text{g}}i}} }}{{N_{{{\text{g}} \bullet }} }} \, } \right. - \left. {\frac{{N_{{{\bar{\text{g}}}i}} }}{{N_{{{\bar{\text{g}}} \bullet }} }}} \right|}$$

where *N*_gi_ refers to the population of the ethnic group *g* of interest in locality i; ${\bar{\text{g}}}$ means the rest of the population or the reference group; and the summation over an index is represented by the dot symbol. A general guide suggests that values of ID less than 30 indicate low segregation, 30–60 indicate moderate segregation, and values over 60 indicate high segregation (Massey and Denton 1993), although of course this varies between national and local contexts.

Residential exposure is computed using P*, which is used to measure the degree of potential contact between members of the same ethnic group. The interpretation of this index is also as straightforward as a percentage. If the index is close to 0, it indicates that the probability of contact or interaction between two members of the same group is very low, whereas if the index values are close to 100, it highlights a high likelihood of sharing the same neighbourhood for two members of the same ethnic group. P* is conceived as a measure of isolation (from people of a different ethnic group) and can be expressed as follows, with $N_{{ \bullet {\text{i}}}}$ being the total population in locality *i*:

2 $$P* = \sum\limits_{i} {\left( {\frac{{N_{{{\text{g}}i}} }}{{N_{{{\text{g}} \bullet }} }}} \right) \, \left( {\frac{{N_{{{\text{g}}i \, }} }}{{N_{ \bullet i} }}} \right)}$$

In this paper, ethnic residential segregation across wards in England and Wales is analysed by age groups and for different age cohorts. Using age is informative because it denotes two important characteristics about an individual: their place in the life cycle—whether a young adult, middle-aged or older—and their membership in a cohort of individuals who were born at a similar time. However, while the characteristics of younger or older adults may differ at a given period, the use of age cohorts allows us to examine the *trajectory* of residential segregation of ethnic groups as age cohorts pass through life course phases, including when household sizes may be growing or reducing.

The computation of ethnic residential segregation with an age dimension is similar to the summary or global calculation for all groups, although yields an index value for each age group. Since index values can be highly sensitive where the group size is small (Voas and Williamson 2000), the analysis focuses on ages 0–64 using 5-year age groups (13 in total), where minority ethnic groups represent a substantial percentage of the total population.^6^

**Fig. 3:**
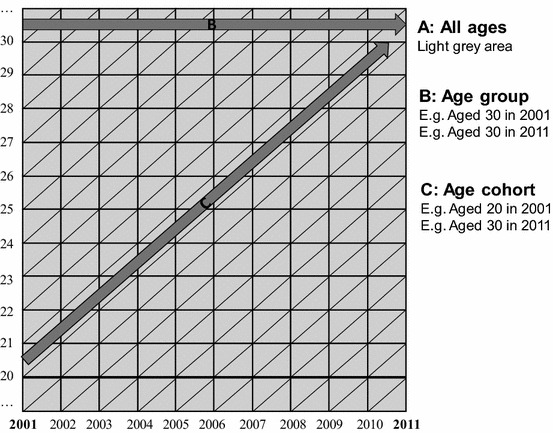
Diagrammatic example of segregation patterns over time for all ages (global), one age group and one age cohort

The construction of age cohorts from cross-sectional data provides a useful approach that captures the longitudinal qualities of panel analysis. Since a cohort is a temporally defined group of individuals, all of whom enter a population in the same time period, age is commonly used to identify birth cohort membership that links observations across cross-sectional data from two censuses (e.g. 2001 and 2011). In this sense, age cohort analysis applied to common cross-sectional data represents an important form of longitudinal research that can be used by researchers who seek understanding of temporal changes in residential segregation. Figure 3 is a diagrammatic example illustrating how we can study patterns over time cross-sectionally for all ages (A) and for one age group (B) or, alternatively, longitudinally across age cohorts (C). The comparison of age groups and age cohorts is a staple of demographic analysis and, although not in conflict, these two perspectives can lead to different conclusions and are better used in tandem.

While aggregate data cannot be used to trace residential segregation transitions from the *same* individuals (e.g. into and out of wards with different levels of segregation), age cohorts have the analytical convenience of aggregates, while retaining the temporal properties of individuals (Myers 1999). A practical advantage of age cohort change is that they can be constructed from large census datasets to examine the relationship between residential segregation over the life course. Thus, while the age group design (B) allows us to describe common processes that are associated with specific ages or stages in the life course, the age cohort design (C) has the virtue of capturing some of the longitudinal quality of panel data, albeit without the fine temporal scale and detailed individual observations. Nonetheless, the compensating advantage of census cohort studies over studies using panel data is that they can cover all geographical locations where individuals reside, and at fine spatial scale.

Given that the cohorts used are synthetic—based on age groups rather than groups of the same individuals at two time points—the populations of the age groups in 2001 and 2011 have been influenced by the interplay of trends in migration and natural change which combine to produce a diversity of segregation patterns across groups and times which depends on the particular histories of immigration and socio-economic mobility. Although the biases that originate from changing cohort composition affect both cross-sectional and panel data, the analysis of age cohort segregation in this paper focuses attention on the ageing of individual ethnic groups, whose residential evenness (ID) and exposure (P*) extend across periods.

Setting the bounds of age cohorts is a necessary step for this analysis. For this purpose, our analysis compares index values of younger and older age groups who age together over time. For instance, we compare the level of segregation of the resident population aged 20–24 in 2001 with index values for those aged 30–34 in 2011. Similarly, those aged 30–34 in 2001 are compared with the equivalent for those aged 40–44 ten years later. In other words, the analysis shows how residentially segregated people are at two time points, a decade a part, thereby revealing the changing patterns of ethnic segregation as individuals age. This approach allows us to go beyond global segregation measures which capture segregation for the *aggregate* population, and illustrate the importance of changes in the level of segregation for different life stages. For example, people aged 16–19 in 2001 (and 20–24 in 2011) are a first age ‘segment’ whose life stage may be primarily influenced by education and young adult mobility. Similarly, other age segments can be related to life stages of family building, employment, and retirement. Following earlier work by Sabater and Finney (2014), it is hypothesised that these stages are likely to correspond to distinct patterns of segregation with, overall, decreases in segregation during young adulthood (mostly due high levels of mobility at this life stage), then progressive segregation increases during the late 20s and early 30s (mostly due to family building at this life stage), and further increases across mid-life until retirement (mostly due to greater immobility as people age).

## Results

### Age-Specific Segregation: Patterns and Change

**Fig. 4:**
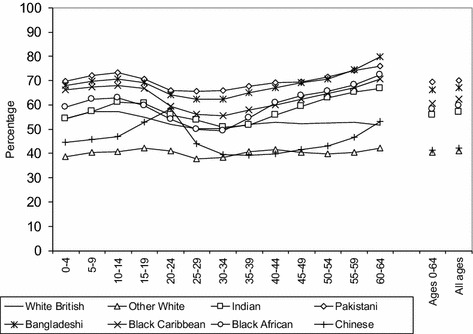
Global and age-specific unevenness (ID) across wards in England and Wales 2011. *Sources*: Own elaboration using data from 2011 Census, Table KS201EW (Crown Copyright)

Our first research question asks whether there are distinctive patterns of segregation for young people compared to those in older ages. This sheds light on the variation in segregation levels within ethnic groups. Figure 4 shows 2011 values of the Index of Dissimilarity (ID); for each of the major ethnic groups ‘global’ (all age group) values are given, as well as values for all aged 0–64 and for each of the 5-year age groups from 0–4 to 60–64 inclusive. The deeper insight into segregation patterns provided through disaggregation into age categories is immediately obvious; distinct levels of unevenness are observed across age groups and between ethnic groups.

Levels of global segregation between ethnic groups are commensurate with other published work exploring segregation at different spatial scales—for districts (Simpson 2012), wards (Catney 2015), lower super output areas (Catney 2017b), and output areas (Catney 2017a). Greater unevenness is observable for the South Asian groups (Bangladeshi, Pakistani and Indian) and two Black groups (Black African and Black Caribbean), with lower unevenness for the Other White and Chinese ethnic groups. The relative positioning of each ethnic group is constant whether all age categories or just those aged up to 64 are analysed, although levels of segregation are slightly lower when the very oldest age categories are omitted.

The results also reveal that this simple approach to analysing segregation by age groups can provide an important contribution to the segregation debate. Most studies interpret segregation as either low, moderate or high, yet this analysis highlights significant differences in segregation by age between ethnic groups. For instance, the oldest age group (60–64) in this study is the most residentially segregated group in 2011, particularly for the Bangladeshi (79.8%), Pakistani (75.8%), Black African (72.5%), Black Caribbean (70.5%) and Indian (66.7%) groups, whose overall segregation can be considered high.

Perhaps more importantly, Fig. 4 also reveals variations in segregation across the life course, represented here by particular age groups and ‘stages’ of life. Three fairly distinctive phases can be identified: higher levels of segregation at (1) the youngest and (2) oldest age categories (those within the 0–19 and 45–64 ranges), and (3) lower levels of segregation for the ‘middle’ age categories (within the 20–44 ranges). While the latter group could be expected to have a level of residential segregation similar to the youngest group, it is important to note that while most children in the youngest group are likely to live in the same household with their parents, not all individuals in the ‘middle’ age categories are parents. In other words, the youngest group becomes more segregated compared to the ‘middle’ age group by virtue of clustering with their immediate family members in the same household and, of course, by a residential locational which is determined by the forces of choice and constraint on parents/families. Hence, the differences that we observe between these two age categories may be interpreted in terms of the impact of household composition and family location on residential segregation. Crucially, these phases are to a large extent common to all ethnic groups, and the consistency in relative levels of segregation found for the global values are generally observable across all age categories. The greatest stability in age-specific segregation patterns can be observed for the ethnic groups Pakistani, Bangladeshi, White British, and Other White. For these groups, there are fewer fluctuations in segregation levels between age categories than for the Chinese, Indian and Black ethnic groups. Indian and Chinese segregation patterns are the most variable across the stages of life analysed.

The most notable deviations from the global trends is the distinctive segregation patterning for the Chinese ethnic group aged 20–24, with a peak of segregation at this age group which is considerably higher than for other age categories for this group (58.6%). No other ethnic group experiences this dramatic a difference in segregation between age categories. A similar, though less intense, spike in unevenness is observed for the oldest age groups for the Chinese population, as is the case for other minority groups. A notable dip in unevenness values is found for the Indian and Black African groups aged 30–34. Pakistani and Bangladeshi segregation levels are fairly consistent across age categories, but with a dip in the 20s and 30s, followed by a relative peak in older ages. The White British is the only group with a story of decreasing segregation in the oldest age groups, with a slight decrease in unevenness at the ages 55–59. It is in the older age categories where the most distinctive ‘clustering’ of values between the highest and lowest values of unevenness can be found; White British, Chinese and Other White segregation is much lower than South Asian and Black segregation for those aged 50 and older.

**Fig. 5:**
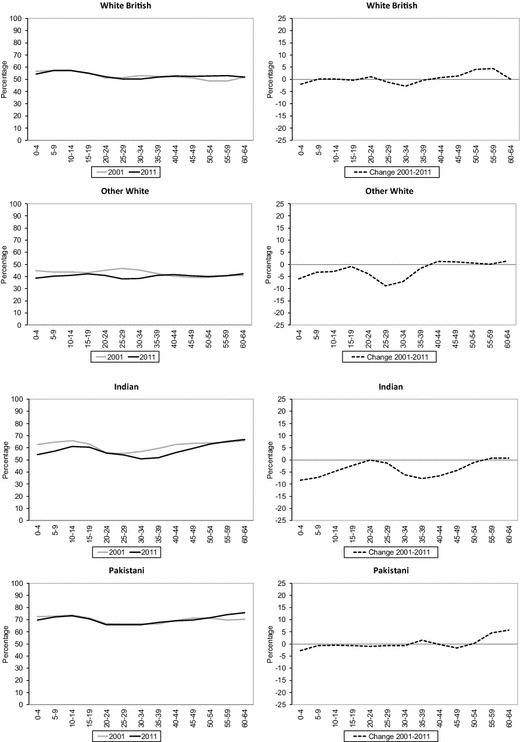

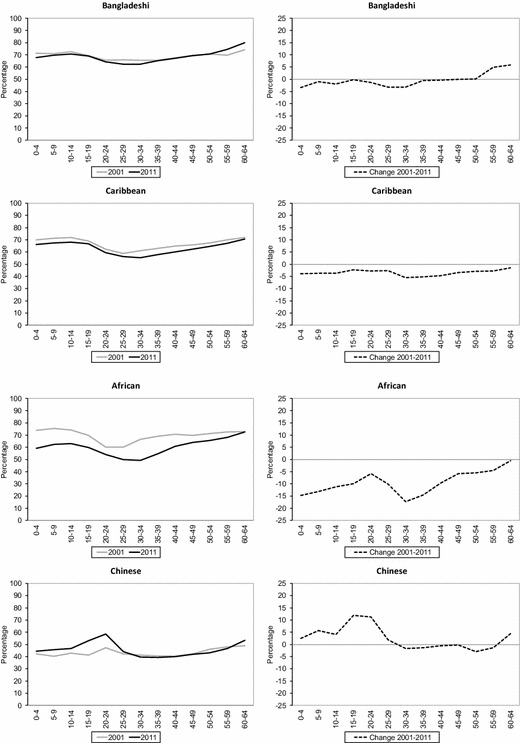
Age-specific unevenness (ID) across wards in England and Wales 2001–2011. *Sources*: Own elaboration using data from 2011 Census, Table KS201EW (Crown Copyright), and complete population estimates based on the 2001 Census (Crown Copyright)

Given that the main pattern indicates higher levels of segregation for the youngest and oldest age categories, and lower levels of segregation for the ‘middle’ age categories for the majority of ethnic groups, we are interested in whether or not these experiences have varied among ethnic groups in the preceding decade across England and Wales. Our second research question therefore asks whether ethnic groups show similar patterns of change in geographical spread and concentration: over time and for each age group and age cohort. To examine this, Fig. 5 shows the level of unevenness (ID) by age groups across wards in England and Wales for 2001 and 2011. Our findings highlight declines over time among all ethnic groups (with the exception of the Chinese group) for the younger groups, and increases for the older age groups.

However, it is clear that not all young age groups have experienced similar levels of decreasing segregation. While notable increases in geographical spread are observed for the youngest and middle age groups for some minority ethnic groups (e.g. Black African, Indian, Other White), for some ethnic groups the decrease in unevenness across younger ages has been more moderate (e.g. Pakistani, Bangladeshi and Black Caribbean). ‘Desegregation’ is particularly noticeable among the Black African group aged 30–34 (from 67 to 49), Indian aged 35–39 (from 59 to 52) and Other White aged 30–34 (from 45 to 38). An increase in unevenness is observed for some age groups for the White British population (e.g. 20–24 and 40 and over), thus (since this measure compares one group to all other groups) indicating decreasing spatial similarity between this group and the rest of the population. While the Chinese group has persistently low levels of segregation between 2001 and 2011, increases in unevenness are noticeable for most young groups (e.g. 0–29).

**Fig. 6:**
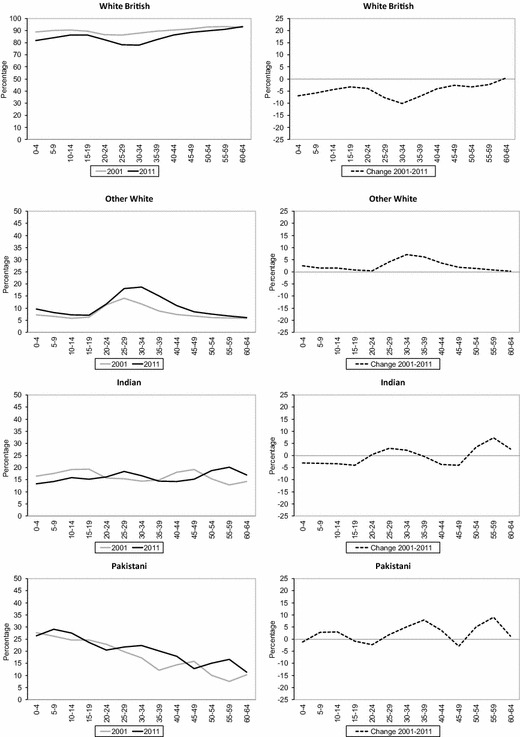

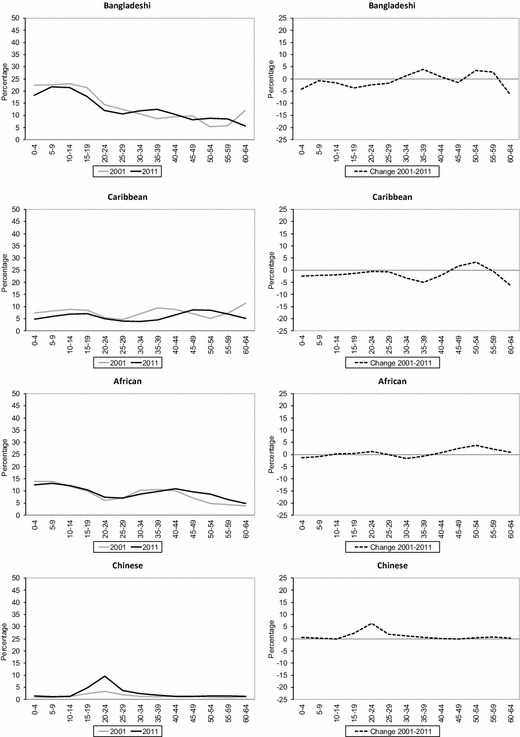
Age-specific exposure (P*) across wards in England and Wales 2001–2011. *Sources*: Own elaboration using data from 2011 Census, Table KS201EW (Crown Copyright), and complete population estimates based on the 2001 Census (Crown Copyright)

Mathematically, if a group’s share of the population rises while ID changes very little, then P* must increase; and the size of the increase depends on the degree to which the group’s share of the population rose over time. P* largely reflects the national composition of ethnic groups across wards (i.e. local average concentration) in England and Wales, and this can be seen in Fig. 6 which depicts the values of age-specific exposure (P*). The values of P* for both 2001 and 2011 show how all ages of the White British group are by far the most exposed compared with other ethnic groups, followed by the South Asian minority groups, particularly the Pakistani group for the youngest and middle-aged groups, and Indian for the oldest age groups. However, the values of P* between 2001 and 2011 for the White British group illustrate that the level of exposure has decreased over the decade for all ages, with the exception of the oldest group (ages 60–64). In contrast, P* values show an increase in exposure for all ages of the Other White group, as well as age-specific increases, mostly for the middle age groups, for the rest of the minority ethnic groups. One of the most notable changes in P* over the decade is an increase in exposure of older groups (e.g. Indian and Pakistani aged 50–64, Bangladeshi aged 50–59, Black Caribbean and Black African aged 50–54).

The values of P* for 2001 and 2011 also demonstrate how across ages all minority ethnic groups are living in wards in which they form a relatively small percentage of the ward’s population. For example, for no age group for any ethnic group does the percentage of that group constitute more than 30% of that ward, indicating that the proportion of a given ethnicity living in high local concentrations is generally low or moderate. The index values of P* are greatest for the Other White (ages 25–34) and Pakistani and Bangladeshi groups, with local concentrations in 2011 that range between 18 and 29%. This implies that, on average, the groups with most exposure to others live in areas where more than 70% of the population are from other groups. These results highlight that there are pronounced age differences in terms of local average concentration of ethnic groups and, as expected, this is reflected in the age-specific measures of residential segregation.

### Residential Segregation by Age Cohorts

The observation that ethnic residential desegregation in the 2000s across age and ethnic groups is marked for young adults lead us to a further inquiry: namely, do younger age cohorts of all ethnic groups represent a cohort/generational change in terms of spatial mixing? To answer our third question, this section focuses not only on period change between 2001 and 2011, but also on the ageing of ethnic groups, as age-specific effects may be compounded by variations in the size of age cohorts entering the population in different periods.

**Fig. 7:**
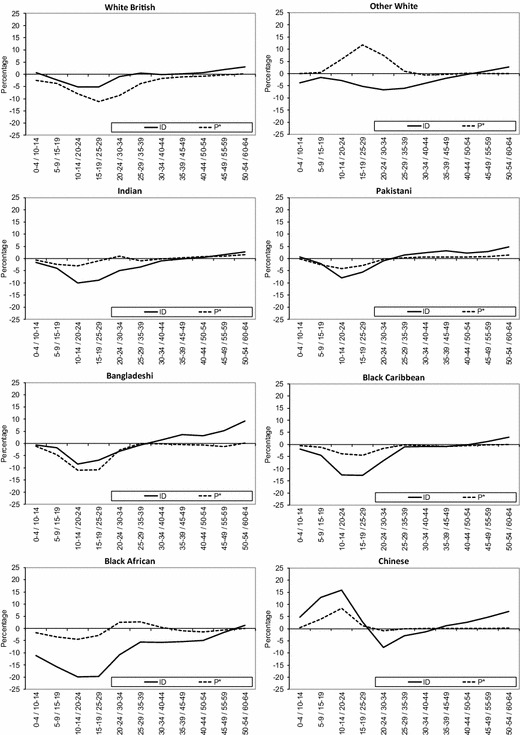
Change in unevenness (ID) and exposure (P*) by age cohorts across wards in England and Wales, 2001–2011. *Sources*: Own elaboration using data from 2011 Census, Table KS201EW (Crown Copyright), and complete population estimates based on the 2001 Census (Crown Copyright)

Figure 7 shows change in segregation across all wards in England and Wales since 2001, in terms of unevenness (ID) and exposure (P*) of ethnic groups by age cohorts. The analysis of ID across age cohorts reveals common changes in geographical spread during the decade for all ethnic groups. First, the youngest age cohort, which refers to children living with their parents, and older age cohorts in their 40s, 50s and 60s, has experienced marginal declines or even small increases in unevenness. Meanwhile, a clear decrease in unevenness is observed among age cohorts in their 20s and 30s in 2011. For instance, ID values show a substantial percentage point decrease for age cohorts 10–14 in 2001 and 20–24 in 2011, particularly among Black African (− 19.9), Black Caribbean (− 12.6), Indian (− 10.1), Bangladeshi (− 8.5) and Pakistani (− 7.9) groups.

The analysis of ID values by age cohorts indicates the existence of a changing experience of ethnic segregation as people age. As would be expected from analyses of residential mobility by age, these results reveal that residential segregation decreases during young adulthood for all age cohorts, then increases during the late 20s and early 30s, and continues to increase across mid-life until retirement. For example, greater unevenness can be observed among age cohorts 35–39 (+0.2), 40–44 (+0.6), 45–49 (+1.9) and 50–54 (+3.1) in 2001 and ten years later for the White British group. A similar pattern of increased segregation is observed among the oldest cohorts (i.e. cohort 50–54 in 2001) of most minority ethnic groups. However, the results also reveal increased unevenness during the decade among younger cohorts for some minority ethnic groups. For instance, a gradual increase in residential segregation can be observed among Pakistani and Bangladeshi age cohorts in their late 20s and early 30s, ranging from +1.4 (Pakistani cohort aged 25–29 and Bangladeshi cohort aged 30–34 in 2001) to +3.6 (Pakistani and Bangladeshi cohorts aged 35–39 in 2001).

Since the most important attributes of age cohorts is the number of people born into the group, the number of arrivals from abroad, and mortality of that group, P* is also employed here to highlight age cohorts differences in population composition between ethnic groups. The larger volume of births, particularly among some groups such as Bangladeshi and Pakistani groups (Kulu and Hannemann 2016), and streams of (family) immigration combine to produce marginal changes in residential segregation for age cohorts in their late 20s and early 30s. However, the most remarkable change in P* over the decade is a decrease for most age cohorts in their teens and 20s. The latter reflects decreases in the average local population due to out-migration from ethnic concentration areas (Finney and Simpson 2009), associated with migration from cities, particularly for those at the family-building life stage.

Finally, the results also suggest some differential trends in the spatial reception of Britain’s most recent immigrant groups. For example, the Other White group (dominated by those with Eastern European origins), despite increasing in size by a factor of nearly two in the course of a decade, has rapidly increased its geographical spread, a situation particularly notable among the middle age cohorts and which are likely driven by labour migration to rural areas (Robinson 2010; Sabater 2015). In contrast, the Chinese group, whose new arrivals are associated with overseas migration to UK universities, highlight an increase in unevenness among the middle age cohorts, particularly in urban centres across Britain. The latter may be associated with post-student retention, which is an important mechanism through which local authorities and private sector organisations ensure that graduates stay put after completing their studies at university (Smith and Sage 2014).

## Discussion and Conclusions

The findings from this study demonstrate the importance of considering age in analyses of segregation patterns and their changes over time. We demonstrate how the analysis of ethnic residential segregation by age groups and the construction of age cohorts from cross-sectional data provides a useful approach for exploring generational shifts in the pattern of life-stage changes in residential segregation. Despite a vast literature on ethnic residential segregation, to date insufficient attention has been paid to age, with only a few studies having investigated the significance of the age dimension in aggregate-level studies of residential segregation (Marcum and Brown 2009; Sabater 2010; Sabater and Finney 2014; Winkler and Johnson 2016). The present study further suggests that residential segregation is a dynamic process with a significant life cycle component, and that commonalities in residential patterning through the life course may transcend differences resulting from ethnicity.

The findings from our analysis are consistent with previous research (Sabater 2010) and add to the evidence base that the dual processes of population growth (within young age groups) and population ageing has been largely accompanied by greater residential integration of ethnic minority groups. We find that for many of the ethnic groups studied residential segregation was lowest for young adults (within the 20–44 ranges) and highest among the younger and (particularly) older groups (within the 55–64 ranges). Moreover, findings from the age group analysis also highlight important similarities in the age patterns of segregation between ethnic groups (with the exception of the Chinese group), thus extending previous ‘global’ understandings of ethnic residential segregation in the UK (Catney 2015, 2016a, 2017a; Harris 2014; Johnston et al. 2013, 2015, 2016a, b; Simpson 2012).

Although the factors involved in the observed decreases in age-specific segregation cannot be disentangled here, studies to date in the UK provide widespread consensus that internal migration, particularly among young adults, is an important contributor to the dispersal of minority ethnic group concentrations (Rees and Butt 2004; Finney and Simpson 2009; Stillwell 2010). Importantly, our results have shown that ethnic residential segregation (as measured by ID) has decreased mostly for those in their 20s and 30s, and this is consistent with the evidence that suggests that internal migration is largely responsible for the changing geography of Britain’s ethnic group populations (Simpson et al. 2008). Thus, despite in situ growth and immigration of young adults can contribute to the expansion of existing clusters of the minority ethnic population, the nature of immigration (e.g. student immigration) and, above all, internal migration (e.g. dispersal from urban concentrations, associated with life course migration) combines to produce increased residential mixing for these ages. Therefore, these results highlight that ethnic groups in the middle-aged phase are more exposed to a greater ethnic mix in their residential environments than older adults.

The findings from the comparison of segregation trajectories for different age cohorts reveal diverse and important aspects of change which are clearly connected to the demography of immigration, the balance of births and deaths, and out-migration from urban clusters. After a decade of rapid decreases in residential segregation among young cohorts, further reductions may be expected in the future if we consider that young cohorts already follow a demographic behavioural pattern which is a function of the events and experiences they have been exposed to earlier in life. Although the patterns of growth of minority populations vary by decade, if the momentum of mixing is generational rather than age-specific, then we would expect to observe greater ethnic mixing among older groups in the future.

Of course, minority ethnic groups in the UK, as elsewhere, have different residential geographies due to the timing and reasons for immigration (Finney 2011). Yet the age cohort analysis utilised here provides insight into a number of dominant trends in the changing patterns of ethnic residential segregation across ethnic groups. First, desegregation of young age cohorts, particularly among ages 10–14, is a reflection of the residential choices and constraints of their parents (for example, out of cities and to suburban or rural locales). Desegregation among the age groups 15–19 and 20–24 are likely reflective of student and graduate mobility, towards higher education or employment. From an age cohort perspective, the latter is a crucial age bracket because it is often the time in which people enter the housing and job markets and, therefore, a moment when young people form their adult lifestyles and begin to make locational choices that may carry into the future.

Second, the widespread residential mixing among young cohorts of every ethnic group suggests that the segregation patterns of young adults differ generationally from those of their parents and grandparents. In the most diverse areas in Britain—generally central urban neighbourhoods—the desegregation of young adults is now widespread; these areas are gaining ethnic minorities (young adults and families/older adults) and White young adults, albeit losing White families/older adults (Sabater and Finney 2014). The observed decrease in young ethnic residential segregation in central urban neighbourhoods is also influenced by a range of non-demographic factors, including the studentification of towns and cities (Smith 2005) and government policies that aim to increase residential land use of brownfield sites in Britain (Bromley et al. 2007). In this sense, the spatial effects of the expansion of higher education in various urban areas, coupled with the new housing developments of towns and cities, appear to be associated with a similar direction of social change. Thus, diverse urban neighbourhoods play an important role both demographically and socially for Britain’s growing minority populations.

Third, our findings indicate that older age cohorts of all ethnic groups experience greater neighbourhood segregation. Many older people, especially those entering pre-retirement ages, may have settled in their neighbourhoods and aged in place. While for many neighbourhood attachment and belonging may have shaped these settlement patterns, for others they may be the outcome of constraint in the housing market. Indeed, although the gradual, if slow, dispersal of all ethnic groups have contributed to desegregation over time, it is important to highlight that exclusionary forces such as racial stereotyping and discrimination have played a crucial role in reinforcing minority ethnic concentration among older cohorts. These have greatly contributed to the current geographies of ethnic groups in Britain, as mutual support between people of similar background was critically important for accessing material necessities, including housing (Phillips and Harrison 2010). From a policy perspective, our findings emphasise how the integration of minority ethnic populations does not take place in a vacuum; rather, they are an expression of the dynamic interplay of societal structuring and the institutional framing of migrants’ (and subsequent generations’) life courses (Wingens et al. 2011).

Further work is needed, however, to address some of the limitations of the study. Although the analyses provide an indication of ethnic residential segregation by age groups and age cohorts across all areas nationally, identifying the significance of spatial relationships between and within ethnic groups, in both urban and rural areas, should be pursued in future studies. More attention is also needed for some ethnic groups (those who face greater inequality and discrimination) and ages (younger and older) in specific locales and regional settings (e.g. Greater London and non-metropolitan areas), particularly where immigration from overseas and/or internal migration has a pronounced effect on age composition, and thus population momentum and housing demand. Since the latter is often regarded as age-related, and the age profile of movers is well established, the mutual dependencies between Britain’s ethnic geography, housing location and residential segregation across the life course clearly requires additional analysis beyond the scope of this study.

The residential patterning for any place is complex and becomes more so when the population is divided by ethnic group and age within a specific context. The findings from this study specifically refer to England and Wales, where the gradual but slow dispersal of all the communities have led to desegregation, particularly for young adults of all ethnic groups. Generally speaking, this trend suggests the overall importance of the assimilation model in the study context, with dispersal from co-ethnic concentration to other parts of the country mostly due to the impact of immigrant growth and the unavailability of housing in original settlement areas. However, the results also reflect other country-specific population dynamics that to a greater or lesser extent have contributed to the population diversification across neighbourhoods. As previously mentioned, in the UK context, the general expectation of university education means a move away from home and moves each year into new accommodation, particularly into university towns, and this is important because the process of studentification, like gentrification, has considerably added to the social-residential mix. While this constitutes further evidence that residential segregation is a context-bound concept (Maloutas and Fujita 2012), it seems clear that by unpacking summary measures of ethnic residential segregation using an age group and age cohort perspective, we improve our understanding of the residential patterning by ethnicity. In turn, we can decompose which age groups and age cohorts have contributed to the overall reduction in segregation observed in other work (Catney 2015, 2016a, 2017a, b; Johnston et al. 2013, 2015, 2016a, b; Harris 2014; Simpson 2007). These insights hint at the processes which shape the patterns of segregation observed, and their changes over time, and allow us to consider the nature of ethnic mixing across the life course.

Traditionally, quantitative studies of segregation have struggled to disentangle the forces of choice and constraint in shaping residential patterning. While beyond the scope of this paper, future work could also usefully explore the interplay between ethnicity and socio-economic status in reinforcing or eroding neighbourhood clustering. The social class—rather than ethnic—selectivity to migration from urban settlement areas (Catney and Simpson 2010) was earlier alluded to, and this selective sorting will play a major role in influencing who mixes with whom. How far is the decreased segregation observed influenced by financial opportunity? Does greater ethnic integration coincide with increased mixing across class lines, and how does this vary between ethnic groups (minority *and* majority)? Related to the theme of this paper, do key phases across the life course produce different residential patterns by ethnicity and socio-economic position? The relationships between ethnic and social segregation are under-explored (Harris et al. 2017), and yet may be crucial in fully understanding the nature of inter-group mixing in residential spaces.

Another unavoidable but important limitation of this study is that that our findings rely on aggregate-level data and, thus, do not follow individual observations over time. While an analysis of regular observations of the same individuals would provide superior insight into the longitudinal dynamics of residential segregation, the geographical focus of panel data in the UK is severely constrained by broad national regions because of sample size limitations. As such, the decennial census is more suitable than panel data for analysis of neighbourhood change. Notwithstanding the limitations of cross-sectional data, the results have underlined the value of taking into account variations in segregation across different ‘stages’ of life, represented here by specific age groups and age cohorts. Examining changing residential experiences as people age using cross-sectional data is considered a time-honoured approach to the longitudinal conception of people’s residential careers (Myers 1999).

Despite these limitations, our study provides a valuable analytical strategy to promote understandings of the processes behind contemporary residential segregation, and the differing experiences of ethnic groups, in a way that avoids over-simplification. While there is a long tradition of studies on residential mobility (Clark 2013; Coulter et al. 2016) and minority internal migration (Finney and Catney 2012) which have emphasised the age-specificity of migration, most work on residential segregation fails to consider the significance of age. Residential segregation is a dynamic process driven by the fundamental changes that occur over a lifetime. Therefore, sharpening our understanding of how ethnic residential segregation plays out across the life course presents us with a crucial opportunity to gain better insight into the residential advantages and disadvantages associated with ethnic group membership, and to help us identify the differences that we observe in terms of life-long residential inequalities.

## Footnotes

1 The data exclude counts of individuals in institutional establishments such as prisons.

2 2001 Census-based data are available online via the UK Data Service, see https://discover.ukdataservice.ac.uk/doi/?sn=6043.

3 2011 Census data are available online via Nomis, see https://www.nomisweb.co.uk/census/2011.

4 For a full description of this approach see Norman et al. (2008).

5 When thinking of the rest of the population we mainly refer to the ethnic mix of an area. The latter is in accordance with policy discussions that emphasise the importance of ‘mixed’ communities in Britain to promote interactions between people from a wide range of ethnic backgrounds, not just in reducing anxiety and prejudice, but also in enabling people to get on better in employment and social mobility (Casey 2016).

6 We do not include small minority ethnic groups at older ages, in post-retirement ages (65 and over).
